# Long Non-coding RNA KCNQ1OT1 Contributes to Antiepileptic Drug Resistance Through the miR-138-5p/ABCB1 Axis *in vitro*

**DOI:** 10.3389/fnins.2019.01358

**Published:** 2019-12-17

**Authors:** Yangmei Xie, Ming Wang, Yiye Shao, Xiaolin Deng, Yinghui Chen

**Affiliations:** ^1^Department of Neurology, Jinshan Hospital, Fudan University, Shanghai, China; ^2^Department of Neurology, Huashan Hospital North, Fudan University, Shanghai, China

**Keywords:** KCNQ1OT1, P-glycoprotein, drug resistance, intractable epilepsy, miR-138-5p, antiepileptic drugs

## Abstract

Compelling evidence has verified that long non-coding RNAs (lncRNAs) play a critical role on drug resistance in various diseases, especially cancer. However, the role of lncRNAs underlying multidrug resistance in epilepsy remains to be clarified. In the present study, we investigated the potential regulatory mechanism of the lncRNA KCNQ1OT1 in regulating antiepileptic drug (AED) resistance in human brain microvascular endothelial cells (HBMECs). The results revealed that expression of P-glycoprotein (P-gp) and KCNQ1OT1 was significantly elevated in phenytoin-resistant HBMECs (HBMEC/PHT). Meanwhile, the activity of nuclear factor-kappa B (NF-κB) was increased in HBMECs/PHT cells. Microarray analysis indicated that miR-138-5p was downregulated in HBMEC/PHT cells. Interestingly, bioinformatics prediction tools indicated miR-138-5p could directly target the transcripts of KCNQ1OT1 and NF-κB p65, and these results were confirmed by luciferase assays. Moreover, KCNQ1OT1 downregulation or miR-138-5p upregulation *in vitro* could inhibit P-gp expression and suppress NF-κB signaling pathway activation. Additionally, knockdown of KCNQ1OT1 or overexpression of miR-138-5p could increase the accumulation of rhodamine 123 (Rh123) and AEDs in HBMEC/PHT cells. Collectively, our results suggested that KCNQ1OT1 contributes to AED resistance through the miR-138-5p/NF-κB/ABCB1 axis in HBMEC/PHT cells, and these results provide a promising therapeutic target for the treatment of medically intractable epilepsy.

## Introduction

Epilepsy is one of the most common neurological disorders, and the main approach to manage epilepsy is still medication therapy. However, approximately one-third of epilepsy cases are resistant to treatment with antiepileptic drugs (AEDs) and develop into medically intractable epilepsy, which has become a major clinical problem in epilepsy therapy (Kwan et al., 2010). The mechanism underlying drug resistance in epilepsy is multifactorial, involving both genetic and environmental factors (Tang et al., 2017). In the past few decades, overexpression of multidrug efflux transporters at the blood–brain barrier (BBB) has been proposed as one of the most significant factors for drug resistance in epilepsy (Potschka, 2010b).

P-glycoprotein (P-gp/ABCB1) is the most investigated member among the multidrug efflux transporters for inducing drug resistance in various cancers (Binkhathlan and Lavasanifar, 2013). In the central nervous system (CNS), P-gp is mainly expressed in the brain microvascular endothelial cells at the BBB, which transports a wide range of diverse chemical agents, including several AEDs such as phenytoin (PHT), phenobarbital (PB), lamotrigine (LTG), and carbamazepine (CBZ) (Zhang et al., 2012). Numerous studies have demonstrated that the expression of P-gp is significantly increased in the brain tissue samples from patients and animal models of refractory epilepsy (Feldmann et al., 2013). It has been accepted that overexpression of P-gp at the BBB contributes to drug resistance in epilepsy by limiting the delivery of therapeutic drugs to epileptic brain tissue. In addition, the use of P-gp inhibitors can enhance the concentration and efficacy of AEDs in the brain tissue (van Vliet et al., 2006). However, there is a limitation of using exogenous P-gp inhibitors in the clinic due to side effects and a lack of tissue specificity. Therefore, identifying endogenous molecules that modulate P-gp expression may provide an alternative strategy.

Long non-coding RNAs (lncRNAs) are endogenous non-coding RNAs longer than 200 nucleotides and control gene expression through a variety of mechanisms, such as acting as protein–DNA or protein–protein scaffolds, competing endogenous RNAs (ceRNAs), and protein decoys, as well as regulators of translation (Quinodoz and Guttman, 2014). The ceRNA hypothesis refers to lncRNAs and other RNA molecules harboring microRNA (miRNA) response elements that can suppress the expression and biological function of each other by competing for common miRNAs, thus regulating miRNA-mediated gene silencing (Salmena et al., 2011). Recently, emerging evidence has indicated that the ceRNA regulation mechanism plays a critical role in drug resistance in various diseases, especially in cancer (Chen et al., 2017). For example, the lncRNA LUCAT1 is upregulated in methotrexate-resistant osteosarcoma cells and modulates P-gp expression by sponging miR-200c (Han and Shi, 2018). Similarly, another study has reported that knockdown of the lncRNA XIST inhibits doxorubicin resistance in colorectal cancer through the miR-124/SGK1 axis (Zhu et al., 2018).

Recently, one study showed that the dysregulated lncRNA KCNQ1OT1 is involved in chemoresistance to paclitaxel in lung cancer cells (Ren et al., 2017). However, the specific mechanism remains to be clarified. Considering the experience in cancer therapy, we aimed to investigate whether KCNQ1OT1 contributes to AED resistance via the ceRNA regulatory mechanism in PHT-resistant human brain microvascular endothelial cells in the present study. Our data indicated that KCNQ1OT1 could contribute to AED resistance in HBMEC/PHT cells through the miR-138-5p/NF-κB/ABCB1 axis, which provides a novel, promising treatment approach for intractable epilepsy.

## Materials and Methods

### Cell Culture and Reagents

Human brain microvascular endothelial cells were purchased from ScienCell Research Laboratories (Carlsbad, CA, United States). The cells were grown in endothelial cell medium (ScienCell, SC-1001) plus 5% fetal bovine serum, penicillin (100 U/ml), streptomycin (10 μg/ml), and 0.03 mg/ml endothelial cell growth supplement in a humidified atmosphere with 5% CO_2_ at 37°C. The conventional therapeutic range for PHT is 10–20 μg/ml. To establish the drug-resistant cell lines, HBMECs were exposed to high doses of PHT (20–40 μg/ml) continuously for 2 weeks according to previous studies (Lu et al., 2004). PB was obtained from Shanghai New Asia Pharmaceutical Co., Ltd. (Shanghai, China). PHT and rhodamine 123 (Rh123) were purchased from Sigma Chemical Co. (St. Louis, MO, United States). PB and Rh123 were dissolved in phosphate-buffered saline (PBS). PHT was dissolved in dimethyl sulfoxide (DMSO).

### MiRNA Microarray

Total RNA containing small RNAs was extracted from cells using TRIzol Reagent (Invitrogen, Carlsbad, CA, United States) and purified with the mirVana miRNA Isolation Kit (Ambion, Austin, TX, United States) according to the manufacturer’s protocol. miRNA profiling was performed using an Agilent miRNA array, which was designed with eight identical arrays per slide, with each array containing 2549 human mature miRNA probes and 2164 Agilent control probes. Each miRNA was detected by probes and was repeated 30 times. Microarray experiments were conducted according to the manufacturer’s instructions. Briefly, total RNA (200 ng) was dephosphorylated and ligated to pCp-Cy3, and the labeled RNA was purified and hybridized to miRNA arrays. Images were scanned with an Agilent microarray scanner (Agilent, CA, United States) and gridded and analyzed using Agilent feature extraction software version 10.10. The miRNA array data were processed for data summarization, normalization, and quality control by using the GeneSpring software V13 (Agilent, CA, United States). To select the differentially expressed genes, we used threshold values of ≥2- and ≤−2-fold change and a Benjamini–Hochberg corrected *p*-value of 0.05. A clustering analysis was performed using Cluster 3.0 and visualized by Tree-View software.

### RNA Interference and Cell Transfection

Small interfering RNA targeting KCNQ1OT1 (si-KCNQ1OT1), an miR-138-5p mimic, and its respective non-targeting sequence (negative control, NC) were synthesized by Gene Pharma Co. (Shanghai, China) and transfected into HBMECs using Lipofectamine RNAiMax Transfection Reagent (Invitrogen, Carlsbad, CA, United States) according to the manufacturer’s instructions. The sequence for siRNA-KCNQ1OT1 was 5′-GCUGGUUACUGGCUUGAAATT-3′. The sequence for the miR-138-5p mimic was 5′-AGCUGGUGUUGUG AAUCAGGCCG-3′.

### Quantitative Real-Time PCR Analysis

Total RNA was isolated from cells using TRIzol Reagent (TaKaRa, Japan) according to the manufacturer’s protocol. Then, cDNA of miRNA was synthesized by the Mir-X miRNA First-Strand Synthesis Kit (TaKaRa, Japan), while the cDNA of mRNA of the target genes, including ABCB1 and KCNQ1OT1, was synthesized by the PrimeScript RT Master Mix (TaKaRa, Japan). Subsequently, the expression of miR-138-5p was detected with the Mir-X miRNA qRT-PCR SYBR Kit (TaKaRa, Japan) in an Applied Biosystem 7300 system (Applied Biosystems, Foster City, CA, United States) using U6 snRNA as an internal quantitative control. The mRNA expression of target genes was measured with a SYBR Premix Ex Taq (Tli RNaseH Plus; TaKaRa, Japan) using β-actin as an internal control.

### Western Blot Analysis

Total protein was extracted from cultured cells with SDS lysis buffer (Beyotime, Shanghai, China) supplemented with 1% phenylmethylsulfonyl fluoride. Equal amounts of protein were analyzed by 10% SDS-polyacrylamide gel electrophoresis and transferred to polyvinylidene difluoride membranes. After being blocked in 5% non-fat milk at room temperature for 1 h, the membranes were incubated at 4°C overnight with the following primary antibodies: anti-P-gp, anti-p-NF-κB p65, anti-NF-κB p65, anti-IκBα, anti-p-IκBα (all from Cell Signaling Technology, United States), and anti-GAPDH (Proteintech Group, United States). The appropriate horseradish peroxidase (HRP)-conjugated secondary antibody or anti-rabbit antibody (Proteintech Group, United States) was incubated with the membranes at room temperature for 2 h. Reactive bands were detected using ECL-Plus (Merck Millipore, Darmstadt, Germany), and the band intensity was quantified using a Bio-Rad 2000 gel imaging system with QUANTITY ONE software (Bio-Rad Laboratories, Hercules, CA, United States).

### Immunofluorescence Staining

For immunofluorescence (IF) staining, cells were seeded onto cover slips in 24-well plates overnight, fixed with 4% paraformaldehyde in PBS for 10 min, washed twice with PBS, and permeabilized with 0.1% Triton X-100 in PBS for 10 min. Fixed cells were preincubated with PBS containing 5% bovine serum albumin (BSA) for 40 min at room temperature. Cells were incubated with a primary antibody (anti-P-gp monoclonal antibody, 1:200 dilution; Santa Cruz Biotechnology) overnight at 4°C. Cells were then stained with an FITC-conjugated secondary antibody for 1 h at 37°C. DAPI (0.1 mg/ml) was added to the secondary antibody mixture to visualize the nuclei. Fluorescence images were collected and analyzed using a confocal laser scanning microscope (Leica, Germany).

### Dual-Luciferase Reporter Assay

The fragments of KCNQ1OT1 containing the predicted binding sites for miR-138-5p were amplified by PCR and then cloned into the pGL3 luciferase promoter vector (Promega, Madison, WI, United States) to construct the KCNQ1OT1-wild-type (KCNQ1OT1-WT) vector. The sequence of the putative binding sites of KCNQ1OT1 was mutated, and the construct was named KCNQ1OT1-mutant-type (KCNQ1OT1-Mut). HEK293T cells were seeded into 24-well plates and were cotransfected with KCNQ1OT1-WT or KCNQ1OT1-Mut and the miR-138-5p mimic or the NC plasmids when the cells reached 50–70% confluence. The cells were lysed, and the luciferase activities were measured 24 h after transfection by a Luc-Pair Duo-Luciferase Assay Kit (GeneCopoeia) according to the manufacturer’s instructions.

### Drug Analysis Using High-Performance Liquid Chromatography

Cell pellets were harvested 48 h after transfection and were washed in PBS. Then, the cells were lysed in methanol and centrifuged at 4°C to obtain the supernatant. The concentration of AEDs was measured by high-performance liquid chromatography (HPLC) with UV detection as described earlier (Shao et al., 2016). In this study, a Waters BEH-C18 (2.1 × 50 mm 1.7 μm) column was used. The mobile phase contained the following: A: water with 0.1% formic acid and B: acetonitrile. The flow rate was 0.4 ml/min, and a gradient was used as follows: 99% A: 1% B (initial), 40% A: 60% B (2 min), 5% A: 95% B (2.1 min), 5% A: 95% B (3.1 min), 99% A: 1% B (3.2 min), and 99% A: 1% B (4.5 min). The column oven temperature was set to 45°C. The chromatography conditions were as follows: negative ESI modes, curtain gas at 45.0 psi, ion spray voltage at 4500 V, and a temperature of 550°C; both ion source gases 1 and 2 were at 45.0 psi, DP-100, EP-10, and CXP-15. The intracellular levels of PB and PHT in the supernatant were quantified by performing a comparison against a standard curve derived from a standard solution of AEDs.

### Rh123 Uptake Assay Using Flow Cytometry

The assay measures P-gp-mediated efflux by detecting the intracellular fluorescence of Rh123 using flow cytometry (Zhang et al., 2013). Briefly, approximately 1 × 10^5^ cells were collected and incubated in a diluted solution with 2 μmol/L Rh123 for 10 min at 37°C. Then, the cells were centrifuged and washed twice in PBS to remove extracellular Rh123. The cells were resuspended and incubated in endothelial cell medium for 1 h. The cells without Rh123 dye served as the negative control. After incubation, the fluorescence of intracellular Rh123 was calculated using a FACSCalibur flow cytometer (Beckman) (excitation/emission wavelength: 485/530 nm).

### Statistical Analysis

Statistical analyses were performed using SPSS 17.0 (SSPS, Inc., Chicago, IL, United States) as well as a one-way analysis of variance (ANOVA) or Pearson correlation analysis. Values are expressed as the mean ± SD, and statistical significance was defined as *p* < 0.05 for all tests.

## Results

### The Expression of KCNQ1OT1 Is Upregulated in PHT-Resistant HBMECs

**FIGURE 1:**
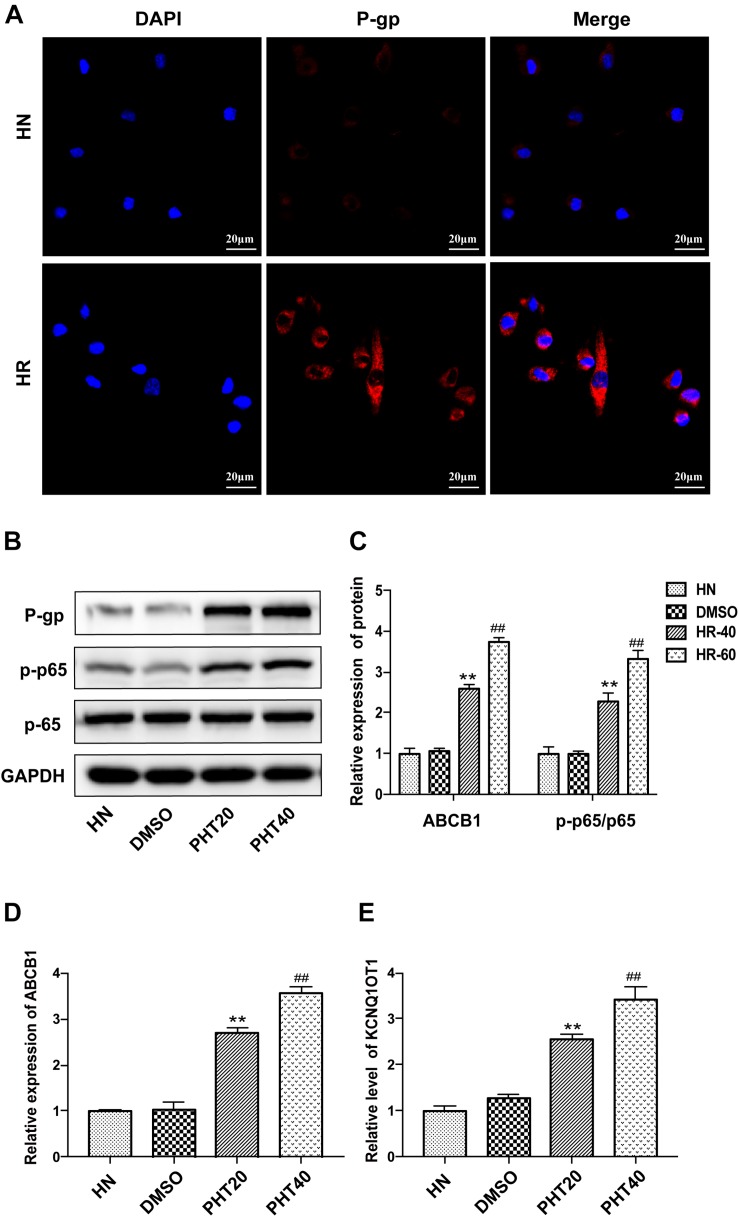
The expression of KCNQ1OT1 was upregulated in HBMEC/PHT cells. **(A)** The expression of P-gp was significantly elevated compared with that in parental cells determined by immunofluorescence staining. **(B,C)** Western blotting revealed that the expression of P-gp and the ratio of p-p65/p65 in HBMEC/PHT cells were elevated compared with those in parental cells. **(D)** The relative expression of ABCB1 was increased in HBMEC/PHT cells compared with that in the corresponding parental cells. **(E)** The relative level of KCNQ1OT1 in HBMEC/PHT cells was elevated compared with that in the corresponding parental cells. (HN stands for HBMEC, HR stands for HBMEC/PHT, HR20 and HR40 stands for HBMECs induced by 20 μg/ml and 40 μg/ml PHT, respectively) (^∗∗^*p* < 0.01 HR20 vs. DMSO, ^##^*p* < 0.01 HR40 vs. DMSO).

The establishment of the PHT-resistant cell line was described in our previous study (Xie et al., 2018). In the present study, it was observed that the expression of P-gp was significantly elevated in HBMEC/PHT cells as determined by IF staining (Figure 1A). The transcription factor nuclear factor-kappa B (NF-κB) has been demonstrated to be one of the key transcriptional regulators of P-gp because NF-κB p65-binding sites exist in the promoter of ABCB1 (Sun et al., 2012; Shi et al., 2015). Most mammalian NF-κB complexes are homo- or heterodimers, and NF-κB p65 is the main functional subunit. Under normal conditions, the NF-κB p65 subunit is sequestered and held inactive in the cytoplasm by inhibitory molecules of the IκB family. In response to multiple stimuli, the IκB molecules can be phosphorylated by IκB kinases, and then NF-κB p65 is phosphorylated (p-p65) and released into the nucleus to drive the expression of downstream target genes. In our study, the ratio of NF-κB p-p65/p65 was observed to be significantly increased in HBMEC/PHT cells, indicating increased activation of the NF-κB signaling pathway (Figures 1B,C). In addition, the expression of ABCB1 and KCNQ1OT1 was increased when HBMECs were induced with different concentrations of PHT (Figures 1D,E). Interestingly, a recent study reported that silencing KCNQ1OT1 could inhibit activation of the NF-κB signaling pathway in cardiac muscle H9c2 cells (Li et al., 2017). Therefore, we speculated that the upregulated level of KCNQ1OT1 may be associated with the increased P-gp expression and NF-κB activation in HBMEC/PHT cells.

### miR-138-5p Directly Binds to the Transcripts of KCNQ1OT1 and NF-κB p65

**FIGURE 2:**
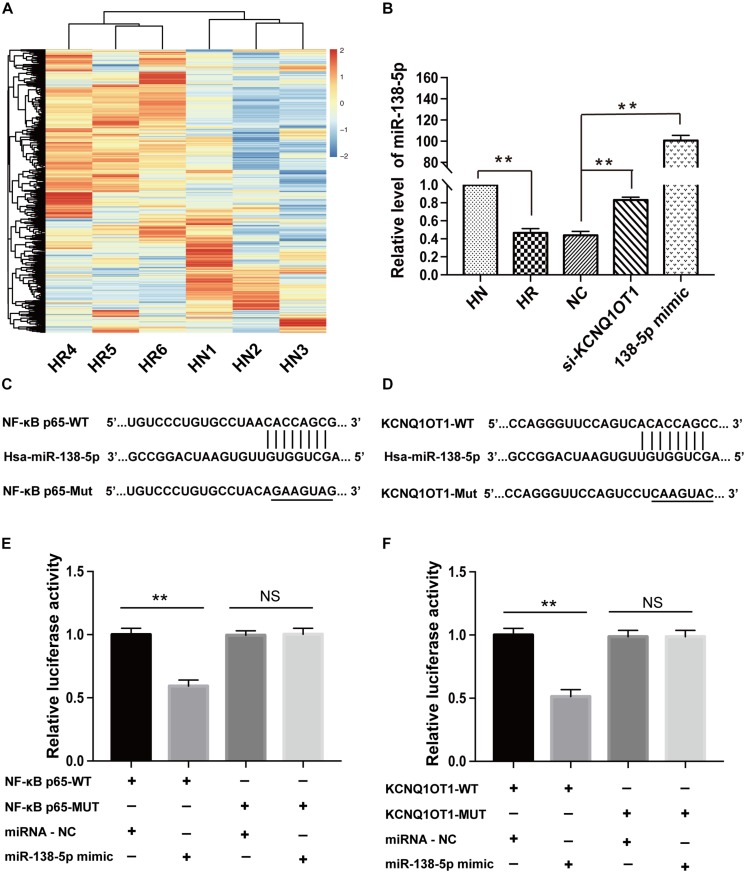
miR-138-5p directly bound to the transcripts of KCNQ1OT1 and NF-κB p65. **(A)** MiRNA expression profiles in HBMEC/PHT cells and their parental HBMECs were detected by the miRNA microarray, and the dysregulated miRNAs are shown in the form of a heat map. (Screening criteria for miRNAs were as follows: *P* ≤ 0.05 and fold change >2.0. Blue or red color on the heat map indicates a decrease or increase in miRNA levels, respectively, and color intensities correspond to relative signal levels on a logarithmic scale.) **(B)** The relative level of miR-138-5p was significantly lower in HBMEC/PHT cells than in the parental cells but was higher in HBMEC/PHT cells transfected with si-KCNQ1OT1 or miR-138-5p mimic than in those transfected with the NC. **(C)** KCNQ1OT1 harbored a putative miR-138-5p binding site, and the designed mutant sequences were indicated. **(D)** NF-κB p65 harbored a putative miR-138-5p binding site, and the designed mutant sequences were indicated. **(E)** Luciferase activity was detected using the dual-luciferase reporter assay. The results showed that luciferase activity was significantly suppressed when cells were cotransfected with KCNQ1OT1-WT and the miR-138-5p mimic. However, there was no statistical effect on luciferase activity when the cells were cotransfected with KCNQ1OT1-Mut and the miR-138-5p mimics. **(F)** Luciferase activity was significantly suppressed when cells were cotransfected with NF-κB p65-WT and the miR-138-5p mimic. However, there was no statistical effect on the luciferase activity when the cells were cotransfected with NF-κB p65-Mut and the miR-138-5p mimic (^∗∗^*p* < 0.01; NS, no significance).

To investigate whether KCNQ1OT1 contributes to AED resistance via a ceRNA regulatory mechanism, microarray analysis was conducted to explore the expression profiles of miRNAs related to drug resistance in HBMEC/PHT cells. Hierarchical clustering showed systematic variations of differentially expressed miRNAs between the PHT-sensitive and PHT-resistant HBMECs (Figure 2A). A total of 92 differentially expressed miRNAs were found, of which 5 were downregulated and 87 were upregulated. Among them, the level of miR-138-5p was confirmed to be downregulated by 50% in HBMEC/PHT cells but upregulated in HBMEC/PHT cells transfected with si-KCNQ1OT1 (Figure 2B). Interestingly, KCNQ1OT1 and NF-κB p65 harbored putative binding sites of miR-138-5p according to the StarBase2.0 bioinformatics database^1^. Luciferase assays were used to confirm the functional binding sites among KCNQ1OT1, NF-κB p65, and miR-138-5p. The binding sites and the designed mutated sites are shown in Figures 2C,D. As shown in Figure 2E, the luciferase activity in the KCNQ1OT1-WT + miR-138-5p mimic group was significantly reduced compared with that in the NC group (*P* < 0.05). However, the luciferase activity of cells cotransfected with the miR-138-5p mimic and KCNQ1OT1-Mut was not significantly different from that of miR-NC-transfected cells. Similarly, luciferase activity significantly decreased in the NF-κB p65-WT + miR-138-5p mimic group but not in the NF-κB p65-Mut + miR-138-5p mimic group. Therefore, our data indicated that miR-138-5p directly binds to the transcripts of KCNQ1OT1 and NF-κB p65 (Figure 2F).

### KCNQ1OT1 Regulates P-gp/ABCB1 Expression Through the NF-κB Signaling Pathway by Sponging miR-138-5p

**FIGURE 3:**
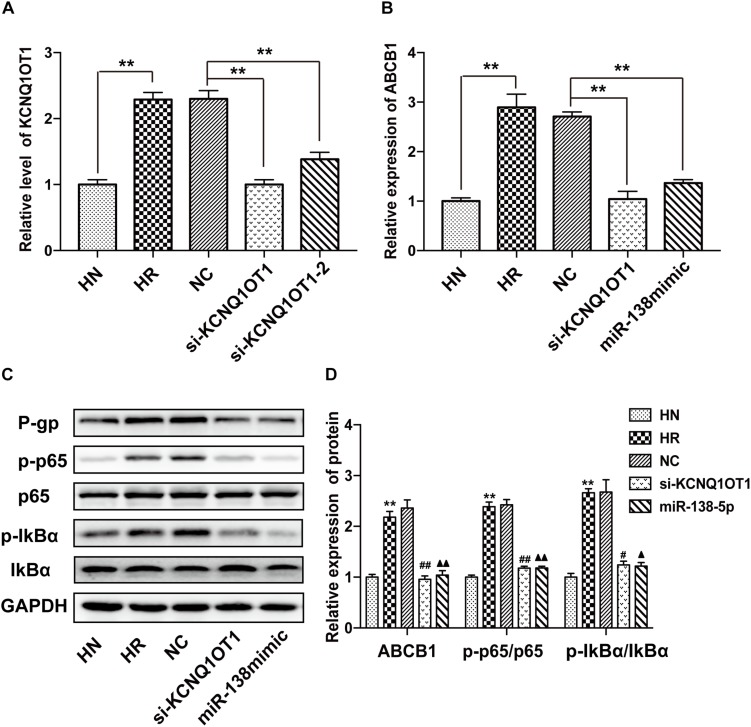
KCNQ1OT1 regulated P-gp expression and affected the NF-κB signaling pathway by sponging miR-138-5p. **(A)** The relative level of KCNQ1OT1 in HBMEC/PHT cells was elevated compared with that in the corresponding parental cells but was reduced in HBMEC/PHT cells transfected with si-KCNQ1OT1 or the miR-138-5p mimic. **(B)** The expression of ABCB1 in HBMEC/PHT cells was upregulated compared with that in the corresponding parental cells but was downregulated after KCNQ1OT1 knockdown or miR-138-5p overexpression in HBMEC/PHT cells. **(C,D)** Western blotting revealed that the expression of P-gp and the activity of key molecules in the NF-κB signaling pathway (the ratio of p-p65/p65 and p-IkBα/IkBα) was elevated in HBMEC/PHT cells compared with that in the parental cells but was reduced after KCNQ1OT1 knockdown or miR-138-5p compared with the NC (^∗∗^*p* < 0.01 vs. HN, ^#^*p* < 0.05 vs. NC, ^##^*p* < 0.01 vs. NC, ▲*p* < 0.05 vs. NC, ▲▲*p* < 0.01 vs. NC).

In accordance with previous data, the level of KCNQ1OT1 was increased in the HBMEC/PHT cells but obviously decreased in HBMEC/PHT treated with si-KCNQ1OT1 (Figure 3A). In addition, knockdown of KCNQ1OT1 or overexpression of miR-138-5p markedly suppressed ABCB1 expression (Figure 3B). To investigate the regulatory effects of KCNQ1OT1 on the NF-κB signaling pathway, we detected key molecules associated with the NF-κB signaling pathway. As shown in Figures 3C,D, the expression ratios of NF-κB p-p65/p65 and the upstream molecules p-IkBα/IkBα in HBMEC/PHT cells were increased significantly compared with those of their parental counterparts. However, knockdown of KCNQ1OT1 or overexpression of miR-138-5p suppressed the activation of the NF-κB signaling pathway as well as P-gp expression. Together with the results of luciferase assays, these findings indicated that KCNQ1OT1 could modulate P-gp expression through the NF-κB signaling pathway by sponging miR-138-5p.

### Downregulation of KCNQ1OT1 or Overexpression of miR-138-5p Increases the Accumulation of Drugs in PHT-Resistant HBMECs

**FIGURE 4:**
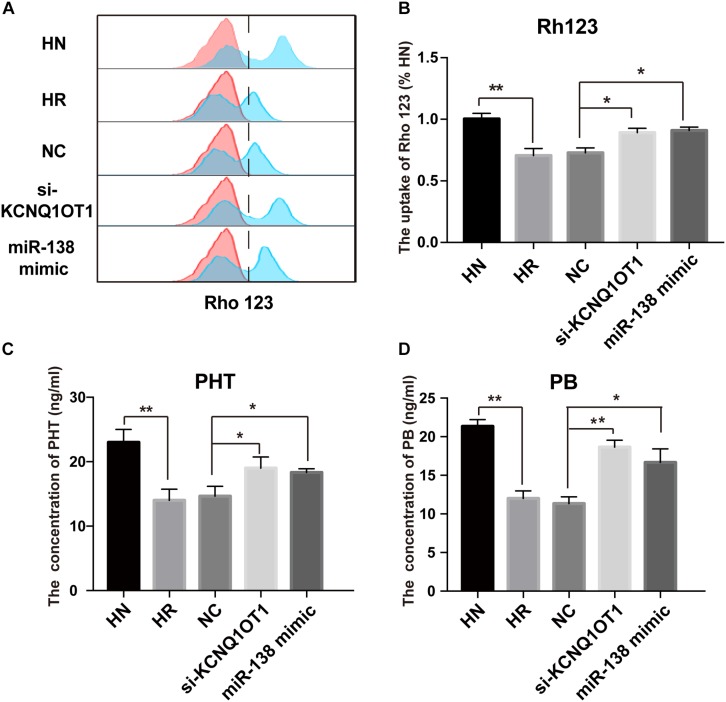
Regulatory effect of KCNQ1OT1 and miR-138-5p on intracellular accumulation of P-gp substrates in HBMEC/PHT cells. **(A,B)** The intracellular uptake of Rh123 was determined by flow cytometry. The results showed that Rh123 content was obviously lower in HBMEC/PHT cells than in the parental counterparts but was increased after KCNQ1OT1 knockdown or miR-138-5p overexpression compared with that in the NC. **(C,D)** The intracellular concentration of phenobarbital (PB) and phenytoin (PHT) was detected by HPLC. The intracellular concentration of PB and PHT in HBMEC/PHT cells was obviously decreased compared with that in the parental counterparts but was increased after KCNQ1OT1 knockdown or miR-138-5p overexpression compared with that in the NC (^∗^*p* < 0.05, ^∗∗^*p* < 0.01).

Accumulating data have indicated that overexpression of P-gp at the BBB is an important factor for the treatment failure of epilepsy by limiting the delivery of AEDs to epileptic brain tissue (Luna-Tortos et al., 2008). Rh123 is a classical substrate transported by P-gp and is commonly used to detect P-gp activity due to its intensive fluorescence (Fontaine et al., 1996). As shown in Figures 4A,B, the Rh123 content was obviously lower in HBMEC/PHT cells than the parental counterparts. However, downregulation of KCNQ1OT1 or overexpression of miR-138-5p increased the intracellular accumulation of Rh123. In addition, different concentrations of PB (20 μg/ml) and PHT (10 μg/ml) were added to the cultures of HBMEC/PHT cells transfected with the si-KCNQ1OT1 or miR-138-5p mimic to determine whether they can reverse the drug resistance of AEDs by inhibiting P-gp expression. Then, the intracellular concentrations of PB and PHT in HBMEC/PHT cells were detected by HPLC. The results showed that the concentrations of PB and PHT in HBMEC/PHT cells were significantly reduced compared with those in the respective control cell populations. However, downregulation of KCNQ1OT1 or overexpression of miR-138-5p markedly increased the accumulation of PB and PHT in HBMEC/PHT cells (Figures 4C,D). Thus, our study indicated that KCNQ1OT1 may be a promising target to overcome drug resistance to AEDs in epilepsy.

## Discussion

The BBB is the physiological protective gatekeeper of the CNS and maintains brain microenvironment homeostasis (Abbott, 2013). Multidrug efflux transporters localized in the brain capillaries of the BBB directly contribute to barrier function by regulating exchange of many compounds between blood and brain. However, overexpression of efflux transporters could restrict the entry of therapeutic drugs, thus leading to drug resistance in neurological diseases, such as brain cancer, amyotrophic lateral sclerosis, and epilepsy (van Vliet et al., 2010; Jablonski et al., 2014; Parrish et al., 2015).

P-glycoprotein is one of the most investigated efflux transporters in medically intractable epilepsy due to its broad substrate spectrum. Numerous studies have indicated that the regulation of P-gp expression in epilepsy is complex and involves several pathological alterations, such as inflammation, oxidative stress, ligand-activated nuclear receptors, excessive glutamate release, and intrinsic overexpression (Leandro et al., 2019). In addition, recent *in vitro* and *in vivo* studies have demonstrated that the long-term application of various AEDs, including PHT, PB, and CBZ, can enhance the expression or function of multidrug transporters, especially P-gp (Alvariza et al., 2014; Ke et al., 2019). Consistent with previous findings, our present study suggested that repetitive exposure to a therapeutic concentration of PHT could induce P-gp overexpression in HBMECs. However, several studies reported the opposite results. One *in vitro* study showed that exposure to common AEDs, such as PHT, PB, and CBZ, does not induce P-gp overexpression at clinically relevant concentrations (Ambroziak et al., 2010). The controversy might be attributed to the use of different cell models and screening methods in different studies.

Since overexpression of P-gp at the BBB has become a serious obstacle for delivering drugs into the CNS, emerging efforts have focused on investigating novel strategies to modulate P-gp expression. Convincing evidence indicated that coadministration of P-gp inhibitors, such as verapamil and cyclosporine A, could enhance the efficacy of AEDs and improve seizure control in patients with medically intractable epilepsy (Nicita et al., 2016). Nevertheless, P-gp inhibitors have not been applied in the clinic due to side effects, such as severe bradycardia and hypotension, and lack of tissue-specific effects (Zhang et al., 2012). In addition, multidrug efflux transporters play a critical role in maintaining the stable microenvironment of the brain by protecting the body from endogenous toxicants and xenobiotics. Exogenous inhibitors of P-gp may cause specific hazards because of enhanced distribution and retarded excretion of xenobiotics.

In view of the protective function of P-gp, growing evidence indicated that targeting the regulatory pathways that modulate efflux transporter expression may be a promising strategy. Because it not only preserves the basal transporter function of efflux transporter but also prevents transcriptional activation from inducing P-gp overexpression by pathophysiological or therapeutic factors (Potschka, 2010a). Recent researches indicated that the mechanism underlying P-gp overexpression in epilepsy involves complex signaling events. For example, the Wnt/β-catenin signaling pathway has been validated to be implicated in drug-induced P-gp overexpression (Lim et al., 2008). In addition, several investigations revealed that the glutamate and cyclooxygenase-2 signaling pathways were associated with upregulation of P-gp in epilepsy (Bauer et al., 2008). Particularly, the transcription factor NF-κB has been proved as a master regulator of P-gp expression in brain capillaries because it appears to be a downstream point of convergence at the stress-induced signals including inflammation, epileptic seizures, and oxidative stress (Miller, 2015). In accordance with previous data, our study showed that activation of the NF-κB signaling pathway contributes to P-gp overexpression in HBMEC/PHT cells.

In recent decades, increasing studies have revealed that non-coding RNAs, mainly miRNAs and lncRNAs, contribute to dysfunction of multidrug efflux transporters in various cancers by modulating the critical molecular pathway and protein transcription (Wang et al., 2019). In the beginning, accumulating literature suggested that small endogenous miRNAs modulate tumor chemotherapy resistance through posttranscriptional regulation of multidrug efflux transporters. However, the primary role of miRNAs is to repress gene expression by inducing degradation or translational inhibition of the target mRNAs. This mechanism is considered insufficient to reverse drug resistance in the treatment of various diseases *in vivo* (Si et al., 2019). Unlike miRNAs, lncRNAs have been shown to regulate efflux transporter expression via diverse mechanisms, including gene imprinting, dosage compensation, and transcriptional or posttranscriptional processing. For example, the lncRNA CCAL upregulates the expression of P-gp via the Wnt signaling pathway in colorectal cancer (Ma et al., 2016). Another study demonstrated that the lncRNA MALAT1 contributes to cisplatin resistance and modulates P-gp expression in lung cancer via the STAT3 pathway (Fang et al., 2018). Particularly, the ceRNA regulatory mechanism has been identified as a canonical theory for lncRNAs to participate in the formation of chemotherapy resistance. For example, in breast cancer cells, the lncRNA FTH1P3 promotes P-gp expression and activates paclitaxel resistance by acting as a molecular sponge of miR-206 (Wang et al., 2018).

**FIGURE 5:**
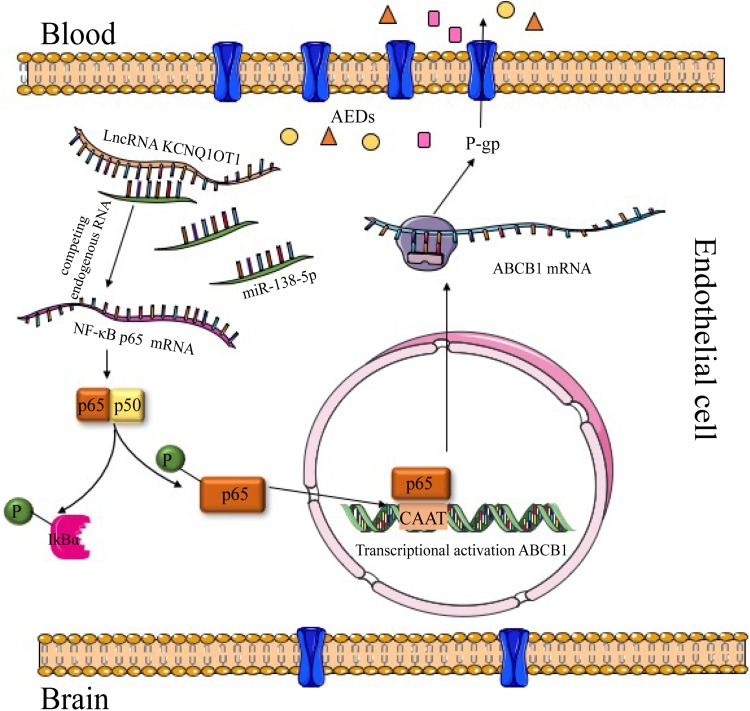
Graph of the potential mechanism of KCNQ1OT1 contributing to AED resistance through the miR-138-5p/ABCB1 axis in intractable epilepsy. In summary, the levels of KCNQ1OT1 and P-gp were elevated in PHT-resistant endothelial cells. KCNQ1OT1 contributed to P-gp/ABCB1 expression by activating the NF-κB signaling pathway as a sponge of miR-138-5p, thus limiting the delivery of AEDs from the blood to the target epileptic tissue.

Considering the necessity to overcome transporter-mediated resistance in cancer therapy, we investigated the role of KCNQ1OT1 in AED resistance *in vitro* and explored the potential mechanism. The expression of KCNQ1OT1 and P-gp as well as the activation of NF-κB in HBMECs were increased when treated with increasing concentrations of PHT. Downregulation of KCNQ1OT1 could inhibit activation of the NF-κB signaling pathway and suppress P-gp expression. The results of microarray analysis and bioinformatics prediction tools revealed that the downregulated miR-138-5p in HBMEC/PHT cells could directly bind to the KCNQ1OT1 and NF-κB p65 transcripts, and this effect was confirmed by luciferase assays. In addition, downregulation of KCNQ1OT1 or upregulation of miR-138-5p could suppress P-gp expression and increase the accumulation of Rh123 and AEDs in HBMEC/PHT cells. Therefore, as shown in Figure 5, our study indicated that KCNQ1OT1 promotes drug resistance to AEDs *in vitro* by targeting the miR-138-5p/NF-κB/ABCB1 axis.

However, there are still some limitations to our study. The present study only investigated the role of KCNQ1OT1 in mediating P-gp expression *in vitro*, and further studies *in vivo* are needed to verify this effect. In addition, the regulatory mechanism of KCNQ1OT1 is complicated and may regulate P-gp expression through multiple targets and pathways. Our study only uncovered the ceRNA regulatory mechanism of KCNQ1OT1, and further investigation is necessary. Although lncRNA-based therapy tends to be powerful drugs to overcome drug resistance, developing drugs targeting lncRNAs is still a great challenge at present. Because lncRNA delivery technologies with increasing bioavailability and decreasing off-target toxicity are urgently needed.

In summary, our study indicated that KCNQ1OT1 promotes drug resistance of AEDs *in vitro* through targeting the miR-138-5p/NF-κB/ABCB1 axis, providing a potential target for overcoming drug resistance in epilepsy.

## Data Availability Statement

The raw data supporting the conclusions of this article will be made available by the authors, without undue reservation, to any qualified researcher.

## Author Contributions

YX performed most of the experiments and wrote the manuscript. YS and XD participated in the statistical analysis. MW gave important intellectual contributions and revised the manuscript. YC conceived the design and supervised the performance of the experiments. All authors have read and approved the final manuscript.

## Conflict of Interest

The authors declare that the research was conducted in the absence of any commercial or financial relationships that could be construed as a potential conflict of interest.

## Footnotes

1 http://starbase.sysu.edu.cn
